# Diminution of Spleen under the Hypodermic Use of Ergot

**Published:** 1876-06

**Authors:** J. H. Miller

**Affiliations:** Moberly, Mo.


Rapid Diminution of a Remarkably Large Spleen under the Hypodermic Employment of Ergot.—*By J. H. Miller, M.D., Moberly, Mo.*—Mrs. S., aged 28, married, the mother of three children, of previous good health, was attacked in August last with bilious fever, as she states, recovered, but shortly relapsed, and has since that time been suffering from frequent attacks of intermittent fever of the quotidian type.

Her physician, not having been penetrated with the incandescence of advancing medical science, but a thoroughly stereotyped routinist, put her through on remedies of the most absolute character, and what her disease had not already done for her he very effectually accomplished.

On the first day of January I called to see her, and found her in extreme prostration and very anæmic, with dyspepsia, neuralgia, and a marked form of intermittent fever, and with a spleen of extraordinary dimensions; so large was it that the whole abdominal cavity, from the extreme left to the middle of the right hypochondrium, and from the umbilicus into the epigastrium, was occupied by its presence.

After improving her general condition by proper alimentation, by tonics, and by stimulants, I addressed my remedies more particularly to the reduction of the size of the spleen, and prescribed for that purpose large doses of quinine, chloride of ammonium and iodine, with iodo-croton liniments externally.

Having failed, after considerable perseverance with these remedies, to elicit a favorable response, I determined to abandon them entirely, and put the therapeutic powers of ergot fairly to the test.

No other remedies were administered except tinc. gentain ℥iii., acidi hydrocyanic dilut. gtt. xxiv., one teaspoonful thrice daily, for some gastric troubles which seemed to call for special attention.

On the sixth day of February I made the first hypodermic injection of ergot. I visited her again on the seventh, and made the second injection; and, although I did not find any diminution in the size of the spleen, yet discovered a very sensible change in its character and consistency. On the 8th I made the third injection, and at that visit there was a very perceptible shrinkage. Owing to professional engagements of a different character I did not visit her on the 9th. On the 10th I made the fourth insertion, and found a very decided diminution in its size; and for four consecutive days I made injections, with rapid and marked diminution. On the 14th it had almost attained its normal size. Thus, from the 9th to the 14th, that enormous spleen had almost entirely disappeared under the hypodermic use of ergot alone. On 14th of March I called, in order to ascertain if the improvement had been permanent. She met me at the door with a recognition of complaisance, and with expressions of profound gratitude. The spleen was normal.—*Medical Record.*

